# Development of a Solid Formulation Containing a Microemulsion of a Novel *Artemisia* Extract with Nematocidal Activity for Oral Administration

**DOI:** 10.3390/pharmaceutics12090873

**Published:** 2020-09-14

**Authors:** Ines Perez-Roman, Filip Kiekens, Damian Cordoba-Diaz, Juan Jose Garcia-Rodriguez, Manuel Cordoba-Diaz

**Affiliations:** 1 Department of Pharmaceutics and Food Technology, Complutense University of Madrid, Pza. Ramón y Cajal s/n, E-28040 Madrid, Spain; inesperezroman@ucm.es (I.P.-R.); damianco@farm.ucm.es (D.C.-D.); 2 Department of Pharmaceutical Sciences, University of Antwerp, Campus Drie Eiken, Universiteitsplein 1, Wilrijk, 2610 Antwerp, Belgium; filip.kiekens@uantwerpen.be; 3 University Institute of Industrial Pharmacy (IUFI), Complutense University of Madrid, Pza. Ramón y Cajal s/n, E-28040 Madrid, Spain; 4 Department of Microbiology and Parasitology, Complutense University of Madrid, Pza. Ramón y Cajal s/n, E-28040 Madrid, Spain; jjgarc01@pdi.ucm.es

**Keywords:** D-optimal mixture designs, silica materials, tablets, solid carrier

## Abstract

Background: Intestinal nematode infections are usually treated with benzimidazole drugs, but the emergence of resistance to these drugs has led to an increasing demand of new anthelmintic strategies. A new microemulsion formulation (ME) consisting of an *Artemisia absinthium* extract with proven nematocidal efficacy was previously developed. The aim of our study is to implement a D-optimal mixture design methodology to increase the amount of a silica material (loaded with this ME) in a tablet formulation, considering its tensile strength and disintegration time. Methods: 16 experiments or combinations of the 6 tablet components (loaded silica, microcrystalline cellulose, polyvinylpyrrolidone, croscarmellose, Syloid^®^ 244 FP and magnesium stearate) were assessed. Tensile strength and disintegration time models were developed, and an optimization process was carried out. Results: Tensile strength was improved by increasing the polyvinylpyrrolidone content, while croscarmellose decreased the disintegration time. The optimized powder mixture contains 49.7% *w*/*w* of the loaded silica material. A compression force of 12 kN was applied to the powder mixture to form tablets with a tensile strength of 2.0 MPa and a disintegration time of 3.8 min. Conclusions: Our results show that D-optimal mixture designs provide a promising approach to formulate liquid-loaded silica materials.

## 1. Introduction

Intestinal nematode infections are usually treated with benzimidazole drugs, but recent studies [1] have evidenced a worrying increase of the cases of resistance to these drugs leading to potential failures of the commonly used therapies. This illustrates the importance of searching for new alternative strategies for the treatment of these kinds of illnesses. As a consequence, medicinal plants have been shown to be a promising source of new anthelmintic treatments [2]. Some species of *Artemisia* have been traditionally used to control this kind of infection; the anthelmintic efficacy of *Artemisia absinthium* has been extensively demonstrated [3]. However, the presence of toxic thujones within the extracts of *Artemisia* constitutes an important drawback for their clinical use. In previous studies, our group developed a new microemulsion (ME) for the vehiculization of thujone-free extracts of *Artemisia absinthium* developed by the Research Institute of Agricultural Sciences (ICA) in Zaragoza (Spain). The original steam distillation extract was previously characterized by GC–MS and the presence of (Z)-epoxyocymene, chrysanthenol, (E)-caryophyllene and linalol as main components was evidenced [4].

Nematocidal activity of the microemulsion containing the *Artemisia* extract was also demonstrated in our previous studies [4,5] using an ex vivo murine model for *Trichinella spiralis larvae*. In fact, the microemulsion system provided a (more than) 95% decrease of the intestinal parasites compared to the original extract. This improvement was also associated with the higher dispersibility of the microemulsion in gastric fluid in comparison to the pure oily extract. This was evidenced through the in vitro dissolution tests, performed according to USP 32 simulating fasting conditions, as described in the same previous study [4].

The adsorption of liquids in solid carriers, transforming them into free-flowing powders, is one of the possible approaches to solidify liquid formulations [6,7]. Solid carriers with a high surface area can be loaded with liquid two or three times their weight without altering their flow properties [6]. Among the materials commonly used in oral formulations [8,9], non-ordered mesoporous silica gel (Syloid^®^ XDP) has shown a good absorption and desorption capacity [10,11,12]. There are two types of Syloid^®^ XDP commercially available, 3050 and 3150, both having a high pore volume (1.7 cm^3^/g) and a high surface area (320 m^2^/g).While Syloid^®^ XDP 3150 has higher average particle size (150 µm) than Syloid^®^ XDP 3050 (50 µm), both are able to retain a high amount of liquid [13,14]. Syloid^®^ XDP 3150 shows better flow properties, improving the content uniformity and the formulation of loaded silica in tablets [15,16].

Tablets are the most widely used solid form, as they improve the dosage of pharmaceutical compounds [17]. However, when silicate powders are compressed into tablets, they usually show low tablet hardness due to their poor compressibility properties. Tablet formulations of liquid-based drug delivery systems also exhibit high disintegration times. One of the reasons is the hydrophobic environment created by some of the components of the liquid formulations that prevents the contact with water molecules and the release of the active substances [18]. As a consequence, the development of tablets based on liquid-loaded silica materials requires the inclusion of binders, but also disintegrants are necessary.

Direct-compression is a tablet manufacturing process with well-known advantages in the pharmaceutical industry, as it requires fewer processing stages and a reduction of the final product cost. However, the powder mixture should have acceptable flow and compression properties in order to obtain tablets with content uniformity and adequate physical characteristics [19,20,21,22].

A direct-compressed tablet formulation based on Syloid^®^ XDP is already published [11], consisting of vitamin E-loaded Syloid^®^ XDP (1:1), microcrystalline cellulose (MCC) PH102, pregelatinized starch, croscarmellose sodium (CCMNa), Syloid^®^ 244 FP and magnesium stearate (MgSt). This powder mixture needs a compression force of 20 kN to produce tablets with adequate properties (hardness: 60 N (tensile strength ≈ 1.6 MPa) and disintegration time: 1 min) [11,16]. However, high compression forces may squeeze the absorbed liquid out of the liquid-loaded silica tablets; therefore, compression forces should be lowered and binders must be incorporated to the powder mixture [23].

Mattsson et al. showed that powder mixtures containing polyvinylpyrrolidone (PVP), as a dry binder, and compressed at 200 MPa had higher axial and radial tensile strength than pregelatinized starch mixtures at the same proportion [24]. These results suggest that PVP may improve the tensile strength of silica tablets, although an efficient disintegrant is required to diminish disintegration times, improving the galenic properties [23].

Response surface methodologies, such as mixture designs, have recently been used in the formulation of microemulsions (ME) [25,26,27], self-emulsifying drug delivery systems (SEDDS) [28,29,30], liposomes [31,32] and tablets [19,33,34,35,36,37,38]. The D-optimal design is one of the most used methodologies in this field, because it allows different composition constraints, in which the sum of all components is constant (100% *w*/*w*). Secondly, it also estimates the excipients’ effects on the formulation characteristics, thus permitting the optimization of the whole system [34,39].

Although ME or SEDDS are already currently used to formulate essential oils with therapeutic properties [40,41], there is increasing interest in loading liquid formulations in solid carriers to improve their doses [12,17] and to obtain the most stable and easy to use solid dosage forms.

In the light of this, the main objective of this work was to focus on the design and optimization of an oral formulation for the treatment of intestinal nematode infections containing an ME of a thujone-free lipidic extract of *Artemisia absinthium* in directly compressible tablets.

Due to the fact that the load of a liquid formulation is usually the limiting step in the development of a solid formulation, the implementation of a D-optimal mixture design to increase the amount of a loaded silica material (e.g., Syloid^®^ XDP 3150: ME) in a tablet formulation is proposed as an interesting tool for the rational and effective development of an acceptable formulation, according to different pharmaco-technical parameters such as tensile strength (as a measure of tablet hardness) or disintegration time.

## 2. Materials and Methods

### 2.1. Materials

The ME was prepared with a surfactant–co-surfactant mixture composed of Tween^®^ 80:propylene glycol (1.5:1 *w*/*w*); both products were purchased from Acofarma Distributions, S.A. (Madrid, Spain). The *Artemisia absinthium* extract (AAE) was provided by the Research Institute of Agricultural Sciences (ICA) (Madrid, Spain). It was obtained by vapor pressure extraction using a stainless-steel industrial distillation plant [38]. The resulting extract is composed by mono- and di-terpene components with promising nematocidal properties [39]. Milli-Q^®^ (Merck-Millipore, Madrid, Spain) water was used throughout all the experiments.

Regarding the solid excipients, PVP K30 (MW 40 kDa) and MCC, Ph. Eur. (Avicel^®^ PH102) were acquired from Merck (Overijse, Belgium). CCMNa, NF (Ac-Di-Sol^®^) was obtained from FMC Health and nutrition (Philadelphia, PA, USA). MgSt Ph.Eur. was purchased from Omega pharma NV (Zwevegem, Belgium). Mesoporous silica gel (Syloid^®^ XDP 3150) and colloidal silicon dioxide (Syloid^®^ 244 FP) were provided by Grace Davison (Worms, Germany).

### 2.2. Methods

#### 2.2.1. Formulation of a Microemulsion Containing the *Artemisia absinthium* Extract

The ME containing the AAE was previously developed and optimized using a D-optimal mixture design, based on its droplet size distribution and conductivity properties. The optimized ME consisted of 66.40% *w*/*w* of the surfactant mixture, 29.35% *w*/*w* of an AAE and 4.25% *w*/*w* of water. The ME formulation was prepared by mixing all the components—at the concentrations previously indicated—under constant stirring for 5 min at 100 rpm using an AGIMATIC-N (Selecta Group, Barcelona, Spain) magnetic stirrer, as described in our previous study [4].

#### 2.2.2. Characterization of the Loaded Syloid^®^ XDP System

Silica material (Syloid^®^ XDP 3150) was loaded with the ME formulation, at a proportion of 1:1.5 *w*/*w* in a beaker with the help of a spatula [18]. Next, the powder mixtures were kept stable for 24 h in a sealed beaker to ensure complete absorption of the liquid formulation in the carrier, according to the methodology proposed by Choudhari et al. [12].

Flow properties of the neat and loaded Syloid^®^ XDP were assessed. Bulk density (ρb) and tapped density (ρt) were determined, as indicated in USP 38 [41], using a Jel Stav 2003 equipment (J. Engelsmann AG, Ludwigshafen am Rhein, Germany). Compressibility index and Hausner ratio were also calculated, using the following equations [42]:

Compressibility index = (100 (ρt − ρb))/ρt(1)

Hausner ratio = ρt/ρb(2)

In order to calculate the angle of repose (α), powder was passed through a funnel placed 10 cm over a plain surface. The funnel was 220 mm high, with a diameter of 212.5 mm; the stem was 122 mm long and it had a diameter of 12 mm. The cone formed by the powder mixture was measured, and the angle was assessed using the following equation [42]:

tan(α) = height/0.5 base(3)

These measurements were repeated three times.

#### 2.2.3. Selection of the Binder, Binder Content and Compression Force

The efficacy of PVP and pregelatinized starch as dry binders was assessed. Based on the previously published formulation, two mixtures were prepared: one included 10% *w*/*w* of PVP and the other, 10% *w*/*w* of pregelatinized starch; the other components were Syloid^®^ XDP:ME (1:1.5) (40.0% *w*/*w*), MCC PH102 (45.5% *w*/*w*), CCMNa (3.0% *w*/*w*), Syloid^®^ 244 FP (1.0% *w*/*w*) and MgSt (0.5% *w*/*w*) [11]. Excipients were combined using a Turbula^®^ T2F (Willy A. Bachofen AG Maschinenfabric, Muttenz, Switzerland) mixer. Mixtures were passed through a 60-mesh sieve. Tablets were made by direct-compression using an EZ20 (Lloyd Instruments Ltd., Bognor Regis, UK) universal testing machine and a 9 mm-diameter round flat die. The compression forces were 10 kN, 12 kN and 15 kN.

Tablets were analyzed for their tensile strength, according to the formula of Fell and Newton [43]:

σ = 2F/πDH(4)

where σ is the tensile strength, F is the breaking force, D is the tablet diameter and H is the tablet thickness. The breaking force of the tablets was determined from the diametral-crushing force, using a PTB411 hardness tester (Pharmatest, Hainburg, Germany). The tensile strength tests were repeated ten times. The binder with better results (considering a tensile strength over 1 MPa as a minimum level of acceptance) was selected for further research [16], and it was included at 10% *w*/*w*, 15% *w*/*w* or 26.5% *w*/*w* content; the powder mixture was compressed at 12 kN or 15 kN.

#### 2.2.4. Optimization of a Tablet Formulation Based on a Syloid^®^ XDP:ME System

The tablet formulation was optimized using a D-optimal mixture design. The independent variables were the amount of loaded carrier and excipients (MCC PH102, CCMNa, Syloid^®^ 244 FP and MgSt), which were set within their composition ranges, to fit a linear, distance-based model (Table 1) [44].

The amounts of the 6 components were varied simultaneously and the whole concentration was adjusted to 100% *w*/*w*; 16 experiments or combinations of the 6 components were assessed. Each experiment was prepared by mixing the components. Powder mixtures were analyzed according to their flow properties. Bulk density and tapped density were measured in the same way as in previous experiments. Then, mixtures were compressed into tablets applying a 12 kN force. All tablet samples were elaborated adjusting the process to obtain the same weight (171 mg).

Tablets were analyzed for their tensile strength and disintegration time. Tests were performed with 6 tablets of each formulation. Disintegration tests were performed using a tablet disintegration tester (Vanderkamp 71b-174B-6; Vankel Industries, INC., Chatham, Ontario, Canada) with 900 mL of distilled water at 37 °C. The disintegration time was expressed as the time (in minutes) in which all tablets disintegrated within a timeframe of 15 min as the limit of acceptance.

Design Expert^®^ (version 7, Stat-Ease Inc, Minneapolis, MN, USA) was used to develop and evaluate the tensile-strength and disintegration-time models. An optimization process of the tablet formulation was carried out in order to find the best compromise between the amount of extract and the tablet properties. The optimal formulation was selected as that having the highest tensile strength and the lowest disintegration time, along with the highest amount of Syloid^®^ XDP:ME.

The optimal formulation assessed by Design Expert^®^ was analyzed in our laboratory, and the statistically predicted results were compared to the actual results, assessing the relative standard error (RSE):

RSE (%) = |((Actual value − Predicted value)/Predicted value)| × 100(5)

## 3. Results

### 3.1. Characterization of the Loaded Syloid^®^ XDP

**Figure 1:**
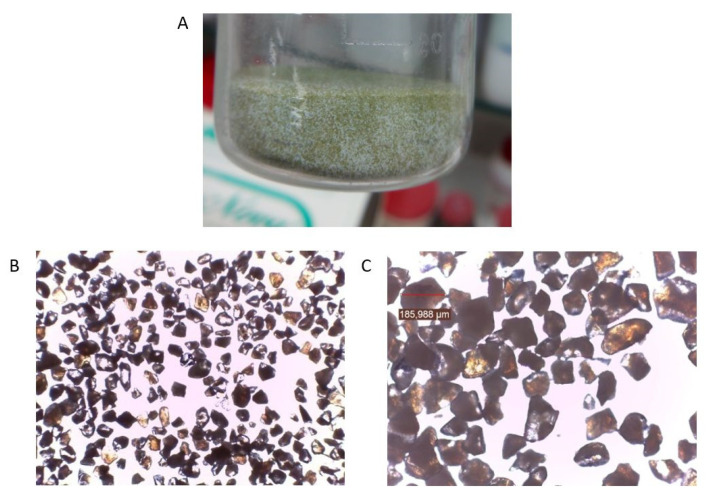
Appearance of the Syloid^®^ XDP:ME (1:1.5) system (**A**) at 40× (**B**) and 100× (**C**).

The Syloid^®^ XDP:ME system showed a free-flowing appearance without any aggregates (Figure 1A). Its morphology was observed using a light microscope at 40× and 100× (Figure 1B,C). Densities of the neat carrier and the loaded system are shown in Table 2. As can be seen, loaded Syloid^®^ XDP showed higher densities (ρb and ρt), compressibility index and Hausner ratio than the neat silica. According to the USP classification [42], flow properties of the neat Syloid^®^ XDP were “Good”, and those of the loaded silica were “Fair”. Therefore, the loaded system could be further formulated into tablets.

### 3.2. Selection of the Binder, Binder Content and Compression Force

**Figure 2:**
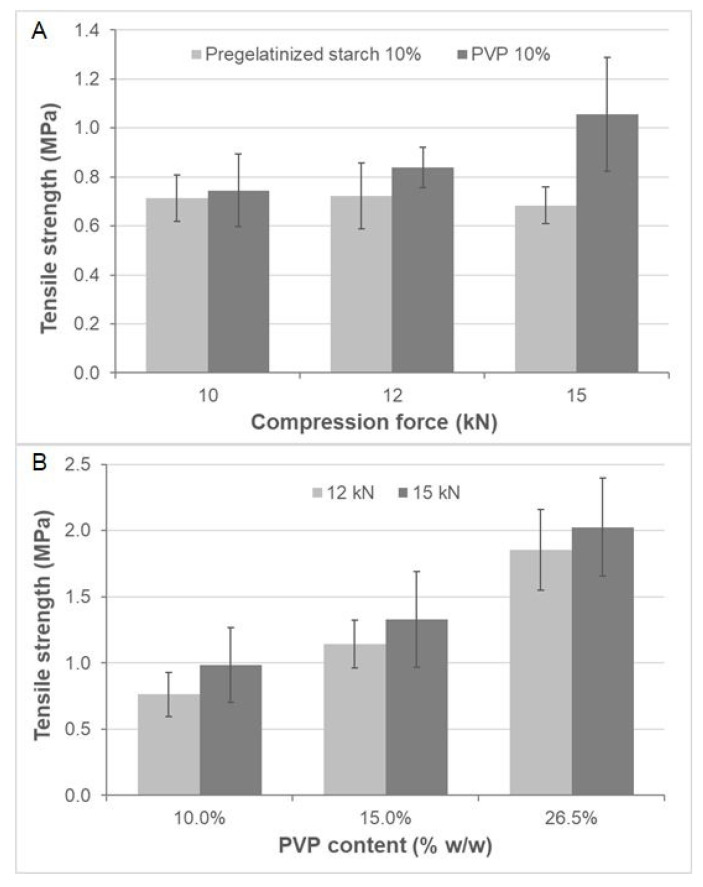
Tensile strength using several binders (**A**) at different compression forces (**B**). Note: average results ± 95% Confidence Interval (CI) (*n* = 3). Abbreviatures: PVP: polyvinylpyrrolidone.

The tensile strength results, depicted in Figure 2, illustrated that tablets containing PVP showed an increase in tensile strength along with the PVP content. However, those changes were hardly seen in pregelatinized-starch tablets, and none of them reached the minimum tensile strength (1 MPa) [16].

As a consequence, the most appropriate binder for further research was PVP. It was included in tablets at 10% *w*/*w*, 15% *w*/*w* and 26.5% *w*/*w* (Figure 2B). The results showed that the tensile strength increased along with the binder content and the compression force. The minimum tensile strength was observed at PVP 10% *w*/*w*, after applying the highest compression force (15 kN). However, a higher amount of PVP (15% *w*/*w*) reduced the compression force required to form tablets with a 1-MPa tensile strength. Therefore, a 15% *w*/*w* amount of PVP and a compression force of 12 kN were selected for further research.

### 3.3. Development of Model Equations

The composition of the experiments and their responses are indicated in Table 3. As can be seen, some of the experiments were performed with the same composition in order to evaluate the model’s accuracy.

The compressibility index of the formulations ranged from 7.69 to 20.45, and the Hausner ratio from 1.06 to 1.28 (Table 4). Most of the formulations had flow properties classified as “Excellent”, “Good” or “Fair” [42]. As could be expected, formulations with low Syloid^®^ 244 content (experiments 10 and 12) had worse flow properties than the other formulations, while experiment 8—with a high percentage of Syloid^®^ 244 (1% *w*/*w*)—showed a “Fair” flow behavior. This may be explained because mixture 8 contained only PVP and the loaded carrier, and no other excipients, such as CCMNa, which may improve flow behavior [44,45]. However, according to the angles of repose, all formulations had an “Excellent” flow behavior [42] and they could be compressed into tablets using a direct-compression method [20].

#### 3.3.1. Tensile Strength

The tensile strength of the powder mixtures after compression varied from 0.14 MPa to 2.70 MPa (Table 5). Since the experiments were assessed based on a linear design, a more complex model could not fit. The linear model showed good performance (*R*^2^ = 0.9158) with low Prediction Residual Sum of Squares (PRESS) (1.75), and a reasonable proximity between predicted *R*^2^ (0.8097) and adjusted R^2^ (0.8690). The adequate precision was 12.34, i.e., greater than 4, which indicates an adequate signal. These results supported the validation of the model to be used for point-prediction [46,47,48].

The characteristics of the linear model are shown in Table 6. As can be seen, F-value was 19.58 (*p*-value = 0.0001), which implies that the model was significant. The closeness of the actual and predicted values to the diagonal line also supported this conclusion (Supplementary data, Figure S1). The normalized probability plot for residuals traced a straight line, indicating that normality assumptions were suggested for the linear model (Supplementary data, Figure S2). However, the lack of fit in the model was significant (*p* = 0.0063), which indicates that the model may not describe the data adequately. This may be due to the interactions among the components of the formulation that could be not elucidated in a linear model.

On the basis of these results, the following equation was obtained:

Tensile strength (MPa) = 0.0094 × Syloid^®^ XDP:ME (1:1.5) + 0.0019 × MCC + 0.1100 × PVP + 0.0058 × CCMNa + (−1.0560) × Syloid^®^ 244 + 1.0220 × MgSt (6)

The magnitude of each coefficient indicates the level of contribution to the response and the positive or negative sign refers to its incremental or decreasing effect on the final response [39]. The resulting equation evidences that the most influential excipients with a positive effect on tensile strength are PVP and MgSt. As a binder, PVP makes connections among the solid particles, improving the hardness of the tablets and, as a consequence, their tensile strength [44,45].

MgSt usually decreases or has no effect on the tensile strength of the tablets [49,50,51,52]. However, our results showed that this excipient increased the tensile strength of the tablets. One of the possible explanations is that the lubricant particles—with a hydrophobic nature—formed bonds with the ME on the surface of the silica material, making the tablets denser, with higher hardness and, consequently, higher tensile strength [53].

Regarding the silica material, our results showed that the loaded Syloid^®^ XDP had a very small but positive influence on the tensile strength of tablets, probably because the presence of the ME system on the surface of the silica particles made them to stick to each other [54]. However, as expected, Syloid^®^ 244 did not form those bonds, so it decreased the tensile strength of tablets [55].

#### 3.3.2. Disintegration Time

The disintegration time of the tablets varied from 0.20 min to 14.00 min (Table 5). The linear model also showed a high correlation ratio (*R*^2^ = 0.9095). The PRESS value (30.98) was higher in comparison to the previous model, and the connection between predicted *R*^2^ (0.6368) and adjusted *R*^2^ (0.8340) was not as close as in the last design. This may be explained because the influence of the excipients on the disintegration time is complex, hence a quadratic design would have a better fit than a linear design. However, the adequate precision (8.200) still validated the model to be used for point-prediction [46,47,48].

The characteristics of the linear model are shown in Table 6. As can be seen, F-value was 12.06 and *p*-value was 0.0044, which implied that the model was significant. The connection between predicted and actual values can be observed in the graph included in Figure S3 in the Supplementary data. The normalized probability plot for residuals traced a straight line, indicating that normality assumptions were suggested for the linear model (Supplementary data, Figure S4).

On the basis of these results, the following equation was obtained:

Disintegration time (min) = −0.0465 × Syloid^®^ XDP:ME (1:1.5) + 0.1038 × MCC + 0.4498 × PVP + (−0.9391) × CCMNa + (−2.2973) × Syloid^®^ 244 + 4.6572 × MgSt (7)

The resulting equation evidences that the excipients that accelerated the disintegration of the tablets were CCMNa and Syloid^®^ 244. CCMNa is a superdisintegrant, so it reduces the disintegration time of the tablet in aqueous environments and therefore accelerates the delivery of the active substances [44,56]. Syloid^®^ 244 also decreased the tablet disintegration time, probably because of its poor tableting behavior [57].

On the other hand, PVP increased the tablet disintegration time, by increasing the tensile strength of the tablets [44,45]. MgSt showed a high positive influence on the disintegration time, maybe due to its lipophilic nature, which reduced the tablet wettability. A reduction in the mixing time of the MgSt may have reduced this influence [20,44,51].

### 3.4. Optimization of a Tablet Formulation

**Figure 3:**
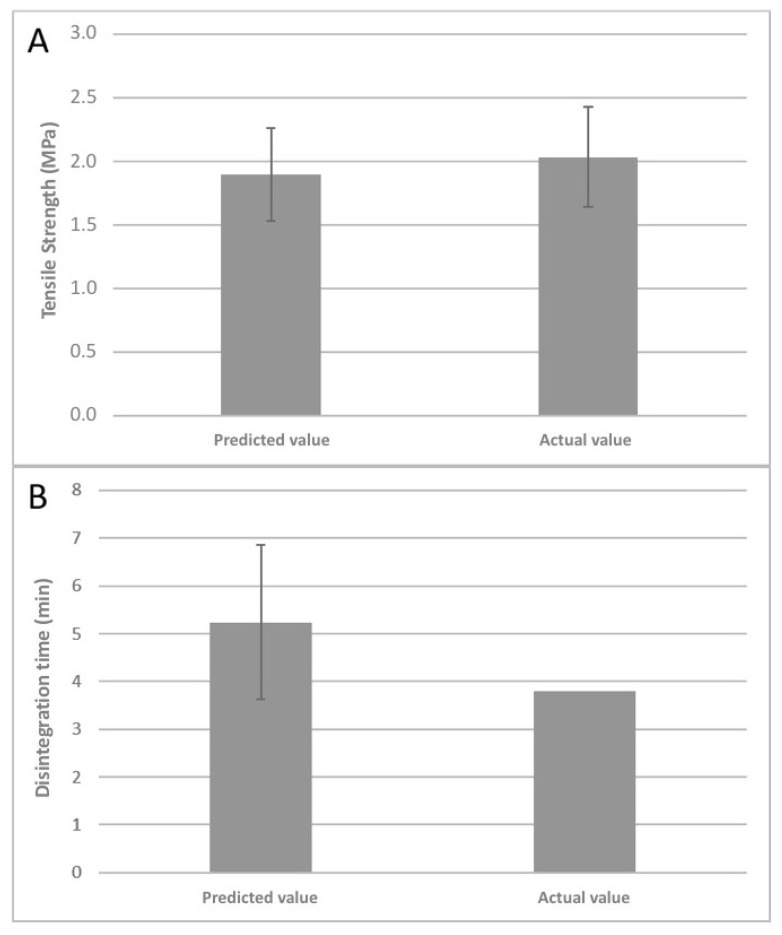
Comparison of predicted and actual values: tensile strength (**A**) and disintegration time (**B**). Note: average results ± 95% CI (*n* = 3).

Previous results were used to optimize the tablet composition. The optimal formulation was composed of Syloid^®^ XDP:ME (1:1.5) (49.71% *w*/*w*), MCC PH102 (33.56% *w*/*w*), PVP (12.55% *w*/*w*), CCMNa (3.03% *w*/*w*), Syloid^®^ 244 FP (0.59% *w*/*w*) and MgSt (0.56% *w*/*w*). The predicted results were 1.89 (95% CI: 1.53–2.26) MPa and 5.24 (95% CI: 3.63–6.85) min for the tensile strength model and the disintegration time model, respectively. The optimal formulation was analyzed in the laboratory, and the actual tensile strength was 2.03 (95% CI: 1.65–2.42) MPa, while the disintegration time was 3.8 min (Figure 3).

There was a close connection between the predicted and the actual results. However, the RSE values were lower in the tensile-strength model (7.46%) than in the disintegration-time model (27.43%). This difference may be explained because the former has a better prediction accuracy than the latter model.

## 4. Discussion

This study shows a methodology to optimize a tablet formulation based on a liquid-loaded carrier, manufactured by direct compression with the main objective of developing a solid dosage form to be used for the treatment of intestinal nematode infections, from an *Artemisia absinthium* extract vehiculized in a microemulsion system.

It includes the following stages: (1) vehiculization of the extract by preparing a microemulsion, (2) loading the liquid formulation (ME) into the solid carrier, (3) selection of the excipients and (4) optimization of the tablet formulation.

In our study, the ME formulation was loaded in the Syloid^®^ XDP in a 1:1.5 *w*/*w* proportion. The loaded silica material had free-flowing properties without any aggregates. These results are consistent with the assessments published by Madhav et al., who loaded 1.61 g of a liquid formulation per gram of Syloid^®^ XDP, resulting in good flow properties [14].

Tablets made by direct-compression methods require the selection of effective excipients with good flow properties and compression behavior [58]. In our study, the selection of excipients was based on the results published by Choudhari et al. [12]. Nevertheless, silica materials have very poor tableting behavior [23] and cannot be exposed to high compression forces that may squeeze the loaded liquid out. Our results showed that powder mixtures including PVP in proportions ranging from 15% *w*/*w* to 26.5% *w*/*w* were able to form tablets showing tensile strength values of 2 MPa, applying a compression force of 12 kN.

D-optimal designs provide a different approach to the traditional formulation procedures applied in the development of tablets, as they quantify the effect of the excipients on the final formulation, under the conditions of the study [59]. They have been previously used to formulate active substances with low flowability and poor tableting behavior [19,35,36,37,38]; however, they have still not been used to develop direct-compressed tablets based on liquid-loaded silica. The main contribution of our study is the use of this methodology to formulate an ME-loaded silica tablet. Due to the poor compressibility properties of the silica material, we selected tensile strength and disintegration time as the main response variables to optimize the new formulation, as they indicate the interparticle bonding and technical characteristics of tablets [60,61]. Results showed that tensile strength was improved by the PVP content, while Syloid^®^ 244 had a negative influence.

PVP increased the disintegration time of the tablets, while CCMNa accelerated this process. It was seen that although the content of PVP in the tablets was higher than the recommended amount (1% *w*/*w*–5% *w*/*w*) [44], it improved the tensile strength at a desirable value [17]. Furthermore, the increase in the disintegration time because of the PVP content was counteracted by the effect of the CCMNa.

An optimal formulation was assessed by the optimization software and analyzed using characterization tests. In order to compare the predicted and the actual results, we calculated the RSE as in other studies [35,36]. However, our RSE values were higher because our experiments were developed using a linear model to analyze the main interactions among the excipients.

Our optimized powder mixture needs a compression force of 12 kN to manufacture tablets with a tensile strength of 2 MPa. Although the compression force cannot be directly compared between different machines [62], we can say that our compression force is lower (12 kN) than the force that Choudhari et al. required (20 kN) to form tablets with a tensile strength of around 1.6 MPa [12]. This may be explained by the higher amount of binder (14.3% *w*/*w*) in comparison to the tablet formulation of Choudhari et al. (10.0% *w*/*w*). As could be expected, higher binder content increases the disintegration time, so our tablets needed more time (3.8 min) than theirs to disintegrate (1 min).

The optimized formulation contains a higher percentage of the loaded silica material (49.7% *w*/*w*) in comparison to the formulation developed by Choudhari et al. (40.0% *w*/*w*) [12]. Moreover, as they loaded the silica material with a 1:1 ratio (Syloid^®^ XDP:vitamin E), the amount of liquid loaded was lower than in our formulation (20% *w*/*w* versus 29.8% *w*/*w* of ME, which implies 8.9% *w*/*w* of AAE).

Previous studies [4] evidenced that the optimized ME showed a W/O structure, but it was capable of dispersing in the gastric environment in less than 15 min, forming 10 nm-sized droplets. Considering that the optimized tablet formulation showed a disintegration time of less than 4 min, the release and dispersion of the ME in the gastric fluid was assured. It was also observed that the droplet size of the ME, once released from the tablet, was similar to the original ME system, what indicated that no incompatibility among absorbed ME and the inert excipients was found, as reported by other authors for these materials [12].

Our results show that D-optimal designs provide a promising tool to improve the formulation of liquid-loaded silica materials in tablets, as it quantifies the effect of the excipients on the properties of the tablets under the conditions of the study. However, due to the limited availability of the *Artemisia absinthium* extract, it was not possible to analyze the interaction of the formulation components. Therefore, more complex designs, such as quadratic models, are needed to develop a more accurate tablet formulation. Those models could probably provide better quality parameters to improve the optimization of the tablet composition. In addition, this new tablet formulation will require further research to study other pharmaco-technical properties like friability and content uniformity. The delivery characteristics of the ME, as well as the nematocidal activity of the natural extract included in these tablets, should also be investigated [63].

## 5. Conclusions

In this study, a D-optimal mixture design was used to improve the amount of liquid-loaded silica material in a tablet formulation. The optimized formulation met the requirements of a tensile strength greater than 1 MPa and a disintegration time lower than 15 min [63,64], providing values of 2 MPa and 3.8 min, respectively, applying a compression force of 12 kN over the mixture, while showing the maximum ME loading capacity with a content of 49.71% *w*/*w* of the loaded-silica materials.

Our results show that D-optimal mixture designs provides a promising option to formulate loaded silica materials with poor compressibility properties. Our model showed good predictability according to the adjusted and predicted *R*^2^ values, as previously described. These results provide a new approach to improve the oral dosage of liquid formulations, especially for those composed of essential oils with therapeutic properties.

## Figures and Tables

**Table 1:** Design constraints for each component.

Component	Minimum (% *w*/*w*)	Maximum (% *w*/*w*)
Syloid^®^ XDP:ME (1:1.5)	32	60
MCC	20	40
PVP	0	20
CCMNa	0	6
Syloid^®^ 244	0	1
MgSt	0.5	1

Abbreviatures: ME: microemulsion; MCC: microcrystalline cellulose; CCMNa: croscarmellose sodium; MgSt: magnesium stearate; PVP: polyvinylpyrrolidone. References: Rowe R.C., 2009 [43].

**Table 2:** Flow and tableting properties of the silica material.

Parameter	Syloid^®^ XDP	Syloid^®^ XDP:ME (1:1.5)
Bulk density (g/mL)	0.281 ± 0.000	0.730 ± 0.029
Tapped density (g/mL)	0.319 ± 0.000	0.882 ± 0.028
Compressibility index	12.000 ± 0.000	17.216 ± 3.291
Hausner ratio	1.136 ± 0.000	1.210 ± 0.049
Angle of repose	26.589 ± 1.142	21.608 ± 2.636

Note: average results ± standard deviation (*n* = 3).

**Table 3:** Composition of the 16 experiments.

Exp	Syloid^®^ XDP:ME (1:1.5) (% *w*/*w*)	MCC (% *w*/*w*)	PVP (% *w*/*w*)	CCMNa (% *w*/*w*)	Syloid^®^ 244 (% *w*/*w*)	MgSt (% *w*/*w*)
1	42.89	29.76	19.41	5.94	1.00	1.00
2	56.53	40.00	0.00	2.19	0.73	0.56
3	52.23	23.09	20.00	3.41	0.66	0.62
4	42.69	39.90	15.67	0.00	0.75	0.99
5	52.23	23.09	20.00	3.41	0.66	0.62
6	36.16	39.67	16.69	6.00	0.66	0.82
7	46.99	40.00	5.10	6.00	0.91	1.00
8	53.92	29.60	14.49	0.00	0.99	1.00
9	55.55	30.94	5.52	6.00	1.00	1.00
10	60.00	24.03	11.74	3.29	0.08	0.86
11	48.58	35.89	11.23	3.03	0.52	0.75
12	60.00	24.03	11.74	3.30	0.08	0.86
13	48.25	30.29	16.10	3.38	0.99	0.99
14	42.69	39.90	15.67	0.00	0.75	0.99
15	56.52	40.00	0.00	2.19	0.73	0.56
16	36.16	39.67	16.70	6.00	0.66	0.82

Abbreviations: CCMNa: croscarmellose sodium, Exp: experiment, MCC: microcrystalline cellulose, ME: microemulsion, MgSt: magnesium stearate.

**Table 4:** Flow properties and responses of the experiments in the D-optimal mixture design.

Exp.	Bulk Density	Tapped Density	Comp. Index	Hausner Ratio	Flow *^,†^	Angle of Repose	Flow *^,‡^
1	0.44 ± 0.00	0.48 ± 0.00	9.78 ± 0.00	1.11 ± 0.00	Excellent	16 ± 2	Excellent
2	0.51 ± 0.00	0.57 ± 0.00	11.11 ± 0.00	1.13 ± 0.00	Good	20 ± 1	Excellent
3	0.42 ± 0.01	0.50 ± 0.02	15.81 ± 2.98	1.19 ± 0.04	Fair	23 ± 3	Excellent
4	0.39 ± 0.02	0.44 ± 0.01	11.00 ± 2.80	1.12 ± 0.04	Good	16 ± 1	Excellent
5	0.42 ± 0.01	0.44 ± 0.14	5.50 ± 2.98	1.06 ± 0.03	Excellent	16 ± 6	Excellent
6	0.36 ± 0.00	0.45 ± 0.00	21.05 ± 0.00	1.28 ± 0.00	Passable	24 ± 3	Excellent
7	0.45 ± 0.00	0.50 ± 0.00	11.11 ± 0.00	1.13 ± 0.00	Good	7 ± 4	Excellent
8	0.33 ± 0.00	0.40 ± 0.00	16.67 ± 0.00	1.20 ± 0.00	Fair	12 ± 5	Excellent
9	0.40 ± 0.00	0.47 ± 0.00	15.00 ± 0.00	1.18 ± 0.00	Good	25 ± 6	Excellent
10	0.37 ± 0.00	0.45 ± 0.00	18.18 ± 0.00	1.22 ± 0.00	Fair	24 ± 1	Excellent
11	0.41 ± 0.00	0.45 ± 0.00	7.69 ± 0.00	1.08 ± 0.00	Excellent	25 ± 2	Excellent
12	0.42 ± 0.00	0.50 ± 0.00	16.60 ± 0.00	1.20 ± 0.00	Fair	24 ± 2	Excellent
13	0.42 ± 0.00	0.48 ± 0.02	12.28 ± 3.04	1.14 ± 0.04	Good	22 ± 1	Excellent
14	0.30 ± 0.00	0.38 ± 0.00	20.46 ± 0.00	1.26 ± 0.00	Passable	25 ± 3	Excellent
15	0.50 ± 0.00	0.57 ± 0.00	12.50 ± 0.00	1.14 ± 0.00	Good	19 ± 0	Excellent
16	0.31 ± 0.00	0.35 ± 0.00	11.54 ± 0.00	1.13 ± 0.00	Good	21 ± 2	Excellent

Notes: average results ± standard deviation (*n* = 3) * Classification according to the USP 38 [41]. ^†^ According to the Comp. Index and Hausner ratio results. ^‡^ According to the results of the angle of repose. Abbreviations: Exp.: experiment.

**Table 5:** Flow properties and responses of the experiments in the D-optimal mixture design.

Experiment	Tensile Strength (MPa)			Disintegration Time (min)
	Minimum Value	Maximum Value	Average ± SD	
1	1.821	2.291	2.052 ± 0.194	5.600
2	0.358	0.420	0.388 ± 0.023	0.300
3	2.257	3.166	2.703 ± 0.401	5.550
4	2.084	2.379	2.230 ± 0.103	14.000
5	2.024	3.016	2.510 ± 0.446	9.217
6	2.570	2.736	2.652 ± 0.030	6.400
7	0.794	0.977	0.884 ± 0.074	1.500
8	1.908	2.497	2.197 ± 0.251	9.417
9	0.000	0.000	0.000 ± 0.000	0.000
10	2.361	3.045	2.697 ± 0.288	5.667
11	0.776	0.779	0.777 ± 0.000	0.000
12	0.133	0.137	0.136 ± 0.000	5.776
13	1.953	3.205	2.567 ± 0.574	7.383
14	2.098	2.487	2.289 ± 0.149	0.383
15	0.412	0.630	0.519 ± 0.099	0.200
16	2.263	2.956	2.603 ± 0.295	6.958

Notes: Results with a 0.000 value indicate that the experiment could not be measured for that variable.

**Table 6:** Results of the linear model based on tensile strength responses.

Parameter	Sum of Squares	Degrees of Freedom	Mean Square	F-Value	*p*-Value
**Tensile Strength (MPa)**					
Model	8.43	5	1.69	19.58	0.0001
Linear mixture	8.43	5	1.69	19.58	0.0001
Residual	0.78	9	0.09	-	-
Lack of fit	0.75	5	0.15	19.86	0.0063
Pure error	0.03	4	0.01	-	-
Corrected Sum of Squares	9.21	14	-	-	-
**Disintegration Time (min)**					
Model	85.09	5	17.02	12.06	0.0044
Linear mixture	85.09	5	17.02	12.06	0.0044
Residual	8.47	6	1.41	-	-
Lack of fit	1.59	3	0.53	0.230	0.8700
Pure error	6.88	3	2.29	-	-
Corrected Sum of Squares	93.56	11	-	-	-

## Supplementary Materials

The following are available online at https://www.mdpi.com/1999-4923/12/9/873/s1, Figure S1: Predicted vs. actual values plot tensile strength model, Figure S2: Normalized probability plot tensile strength model, Figure S3: Predicted vs. actual values plot disintegration time model, Figure S4: Normalized probability plot disintegration time model, Table S1: Final report of the tensile strength model, Table S2: Final report of the disintegration time model.
