# Supplementary material for: Development of a Solid Formulation Containing a Microemulsion of a Novel Artemisia Extract with Nematocidal Activity for Oral Administration
Source: Pharmaceutics. 2020 Sep 14;12(9):873. doi: 10.3390/pharmaceutics12090873 (PMC7559406; doi:10.3390/pharmaceutics12090873)
Supplement: Supplementary file 1 [file pharmaceutics-12-00873-s001.pdf]

pharmaceutics

Supplementary Materials: Development of a Solid Formulation Containing a Microemulsion of a Novel *Artemisia* Extract with Nematocidal Activity for Oral Administration

Ines Perez-Roman, Filip Kiekens, Damian Cordoba-Diaz, Juan Jose Garcia-Rodriguez and Manuel Cordoba-Diaz

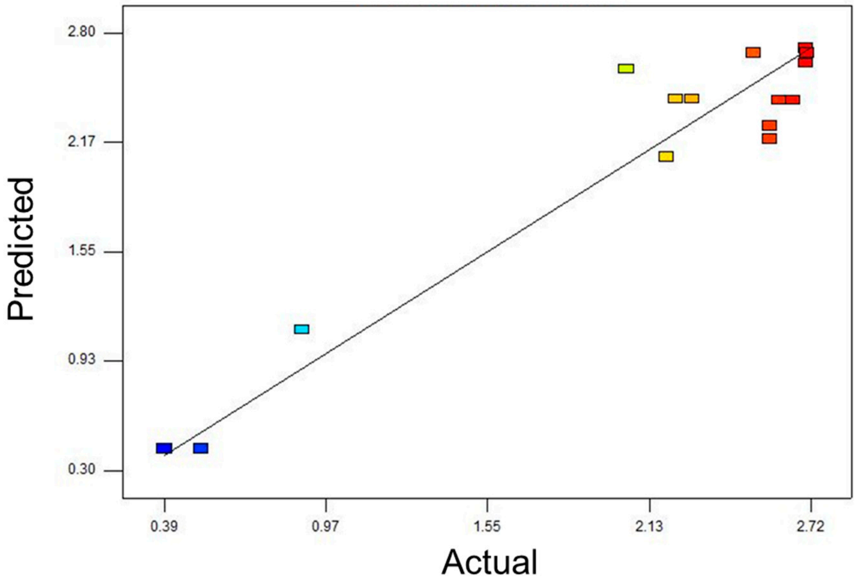

Figure S1. Predicted vs actual values plot tensile strength model.

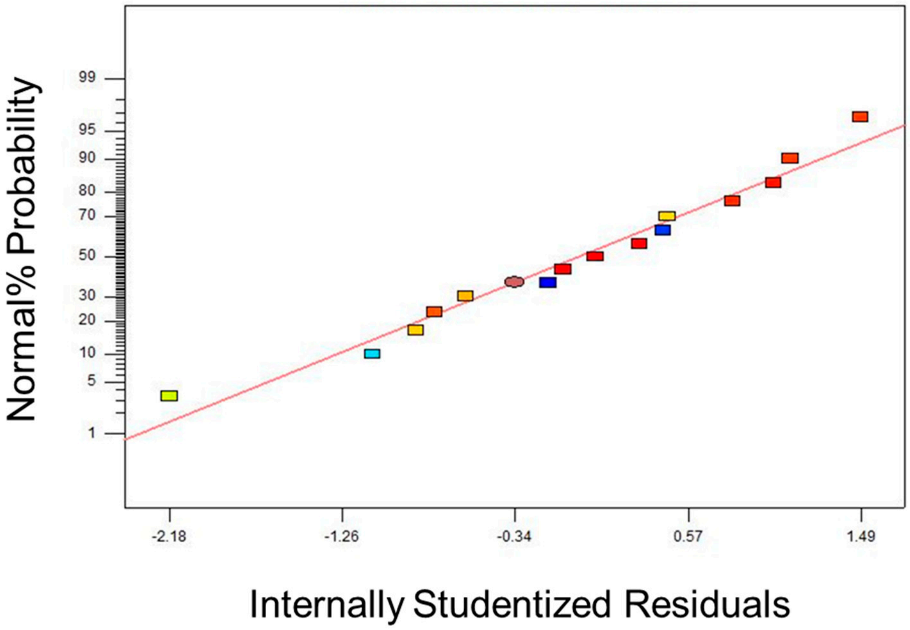

Figure S2. Normalized probability plot tensile strength model. .

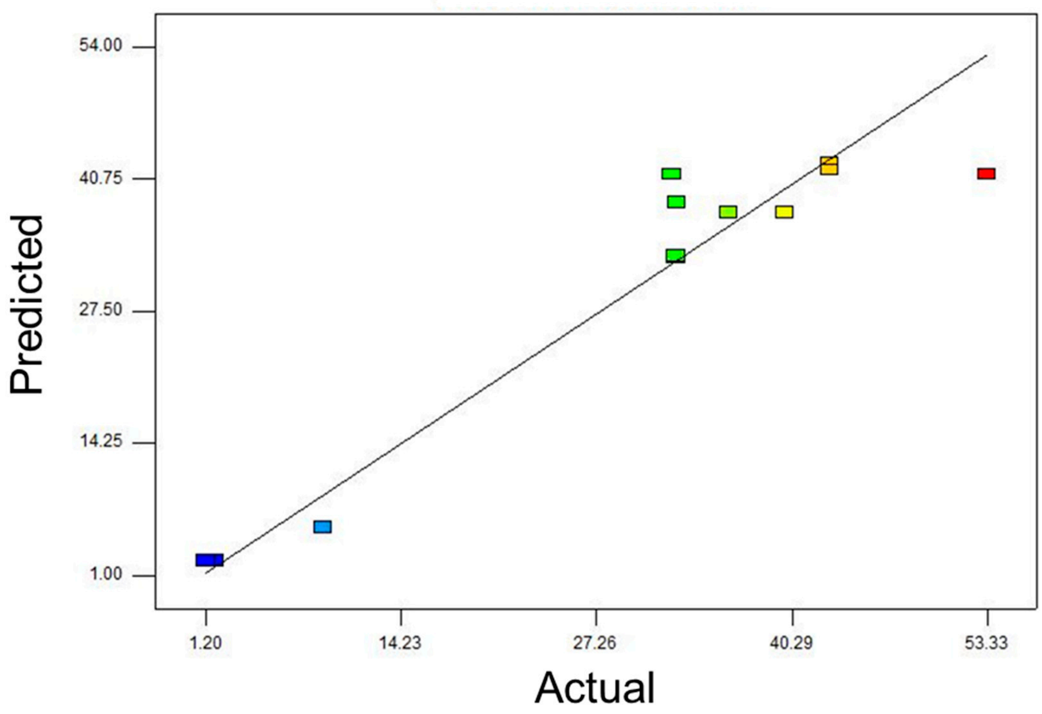

Figure S3. Predicted vs actual values plot disintegration time model.

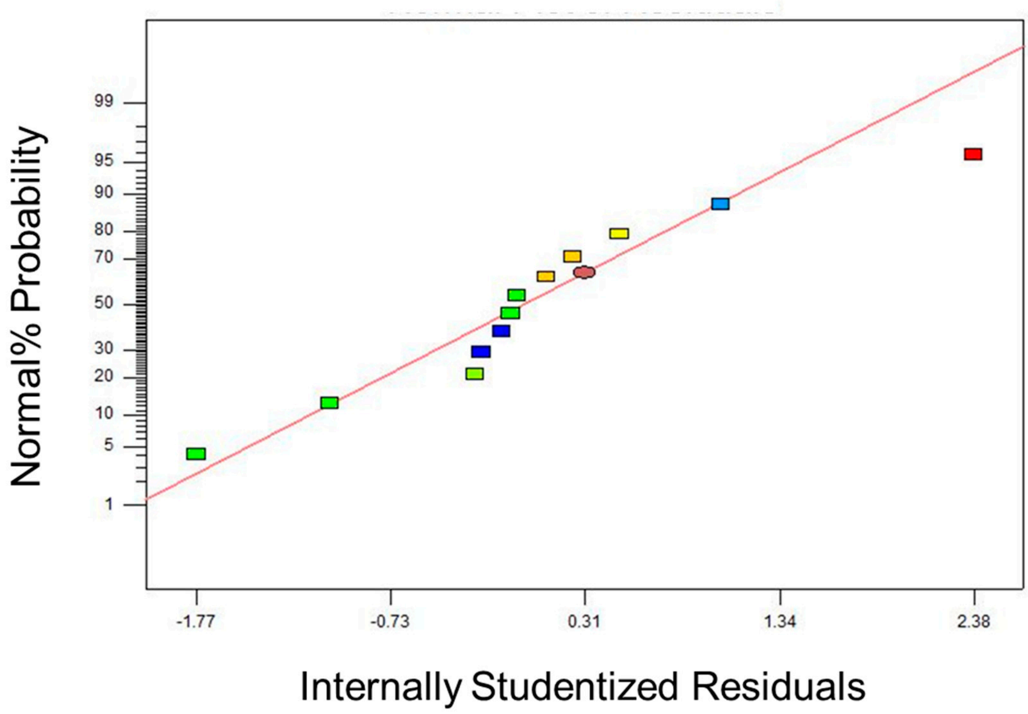

Figure S4. Normalized probability plot disintegration time model. .

Table S1. Final report of the tensile strength model.

Run	Actual Value	Predicted Value	Residual	Leverage	Internally Studentized Residual	Externally Studentized Residual	Influence on Fitted Value (DFFITS)	Cook's Distance
1	2.51	2.68	-0.170	0.404	-0.770	-0.752	-0.619	0.067
2	2.65	2.42	0.230	0.392	1.023	1.027	0.825	0.113
3	0.39	0.43	-0.037	0.464	-0.170	-0.161	-0.150	0.004
4	2.70	2.72	-0.019	0.527	-0.092	-0.087	-0.092	0.002
5	2.05	2.60	-0.540	0.274	-2.175	-2.978	-1.827	0.297
6	2.29	2.42	-0.140	0.418	-0.606	-0.583	-0.495	0.044
7	2.57	2.27	0.300	0.161	1.113	1.130	0.495	0.040
8	0.52	0.43	0.094	0.464	0.437	0.416	0.388	0.028
9	2.70	2.68	0.018	0.404	0.079	0.074	0.061	0.001
10	2.23	2.42	-0.200	0.418	-0.872	-0.859	-0.729	0.091
11	0.88	1.10	-0.220	0.535	-1.101	-1.116	-1.198	0.233
12	2.20	2.09	0.100	0.403	0.463	0.442	0.363	0.024
13	2.57	2.19	0.370	0.270	1.486	1.612	0.980	0.136
14	2.70	2.63	0.067	0.472	0.313	0.296	0.280	0.015
15	2.60	2.42	0.180	0.392	0.809	0.792	0.636	0.070

Table S2. Final report of the disintegration time model.

Run	Actual Value	Predicted Value	Residual	Leverage	Internally Studentized Residual	Externally Studentized Residual	Influence on Fitted Value (DFFITS)	Cook's Distance
1	9.22	7.11	2.10	0.452	2.392	10.15	9.22	0.788
2	6.40	6.59	-0.19	0.494	-0.223	-0.204	-0.202	0.008
3	0.30	0.42	-0.12	0.474	-0.136	-0.124	-0.118	0.003
4	5.67	5.72	-0.049	0.529	-0.061	-0.055	-0.059	0.001
5	5.60	6.61	-1.01	0.360	-1.060	-1.074	-0.805	0.105
6	7.38	7.31	0.077	0.454	0.088	0.080	0.073	0.001
7	0.20	0.42	-0.22	0.474	-0.252	-0.231	-0.219	0.010
8	5.55	7.11	-1.56	0.452	-1.779	-2.362	-2.15	0.436
9	1.50	0.95	0.55	0.799	1.032	1.039	2.07	0.705
10	7.38	7.18	0.20	0.539	0.252	0.231	0.250	0.011
11	5.67	5.74	-0.078	0.477	-0.091	-0.083	-0.079	0.001
12	6.95	6.59	0.36	0.494	0.428	0.397	0.393	0.030
